# The Browning Properties, Antioxidant Activity, and α-Glucosidase Inhibitory Improvement of Aged Oranges (*Citrus sinensis*)

**DOI:** 10.3390/foods13071093

**Published:** 2024-04-02

**Authors:** Ting-Yu Hsu, Kai-Min Yang, Yi-Chan Chiang, Li-Yun Lin, Po-Yuan Chiang

**Affiliations:** 1 Department of Food Science and Biotechnology, National Chung Hsing University, 145 Xingda Road, South Dist., Taichung City 40227, Taiwan; a0961511502@gmail.com (T.-Y.H.); chiangyichan@gmail.com (Y.-C.C.); 2 Department of Food Science, National Quemoy University, 1 University Rd., Jinning Township, Kinmen County 89250, Taiwan; ykmin@nqu.edu.tw; 3 Department of Food Science and Technology, Hungkuang University, No. 1018, Sec. 6, Taiwan Blvd., Shalu Dist., Taichung City 43302, Taiwan; lylin@sunrise.hk.edu.tw

**Keywords:** orange, aging process, solid state fermentation, browning reaction, antioxidant activity, α-glucosidase inhibitory ability

## Abstract

Oranges contain many natural active chemicals, organic acids, and polysaccharides. Aging processing is commonly used to modify the color, quality, functional components, and stability of fruits. This study assesses the preparation of aging black oranges using various pre-treatments and solid fermentation. Oranges were aged for six weeks in fresh, non-blanching, blanching, and hot air-assisted aging cycle (AA) groups. The oranges’ shrinkage ratio, color difference values, and soluble solids content changed significantly (*p* < 0.05). Principal component analysis indicated that aging fermentation treatment accelerated glycolysis and increased the ratio of reducing sugars. The enhanced browning can be associated with the oxidation of ascorbic acid (0.66–0.47 mg/g) and the formation of 5-hydroxymethylfurfural (5-HMF) (0.09 mg/g). Furthermore, the presence of free polyphenols led to an increase in the total polyphenol and total flavonoid content. It also had a synergistic effect with 5-HMF in increasing the 2,2-diphenyl-1-picrylhydrazyl free radical-scavenging capacity and ferric ion-reducing antioxidant power (*p* < 0.05). AA had superior α-glucosidase inhibitory ability increasing from 67.31 to 80.48%. It also reduced the development time by 33%. Therefore, aging technology can enhance the bioactive compounds in oranges and provide a reference for future whole-fruit aging fermentation and health product creation.

## 1. Introduction

The orange, scientifically known as *Citrus sinensis*, belongs to the *Rutaceae* family. It has an appealing scent, with a taste combining sweetness and acidity, and it contains beneficial nutrients. It is a significant fruit globally. The Food and Agriculture Organization of the United Nations’ Corporate Statistical Database (2024) indicates that global citrus production was around 84 million tons in 2022. Oranges contain several biologically active substances, including phytochemicals, polyphenolic compounds, flavonoids, organic acids, and dietary fiber [1]. Some common compounds with various physiological roles are hesperidin, nobiletin, quercetin, rutin, ascorbic acid, and soluble and insoluble dietary fiber. These compounds have antioxidant, anti-inflammatory, anticancer, and cardiovascular protective effects [2,3,4,5].

The United Nations has introduced 17 Sustainable Development Goals (SDGs) to encourage global collaboration in reducing waste generation, promoting crop recycling, and prioritizing the green economy and sustainable material manufacturing [6]. Most oranges are used in the fresh fruit, juice, and jam sectors. Orange peels generated during juice processing and various byproducts are often discarded as waste due to their irregular appearance, over-ripeness, or minor bug infestation, leading to material wastage and significant environmental issues [7]. Orange peel is rich in flavonoids, essential oils, dietary fiber, and polysaccharides (pectin), making it an excellent material for regeneration. It is commonly recycled through processes such as essential oil extraction, dietary fiber production, pectin recovery, and plant enzyme isolation [8,9]. Research on citrus in nutritional healthcare has recently become popular, necessitating the development of processing and value-added technologies to prolong its shelf life.

Aging is a solid-state fermentation process that involves controlling temperature and humidity to expose complete fruits and vegetables (including their skin and flesh) to a sequence of enzyme and chemical reactions, resulting in a dark brown color. It is a green process with no additives and minimal pollution. Vegetables and fruits frequently have browning responses as an aging process, resulting in a deeper color, firmer texture, the development of sweet and sour flavors, and an increase in scent [10,11,12,13,14]. Several sources indicate that aging enhances the antioxidant, anti-allergic, antidiabetic, anti-inflammatory, hypolipidemic, and anticancer characteristics of black garlic, black apple, and black bitter melon [15,16,17,18,19]. Aging technology causes enzymatic and non-enzymatic browning. High temperatures and high humidities prevent enzymatic browning and promote non-enzymatic browning, such as during the Maillard reaction, caramelization, and ascorbic acid degradation [10,11,12]. The Maillard reaction involves the interaction of carbonyl compounds and free amine groups at elevated temperatures. The intermediate compound 5-hydroxymethylfurfural (5-HMF) is produced under acidic conditions, leading to enhanced browning and antioxidant activities [10,11]. Furfural is naturally present in fruits and vegetables. However, its level can increase due to the oxidative degradation of ascorbic acid, particularly under acidic and high-temperature conditions, which can enhance the cleavage reaction [12]. This reaction also greatly decreases the moisture level, leading to structural breakdown, which releases polyphenolic compounds and other active substances from the cell wall while increasing the concentration of free polyphenols [15,19]. Previous studies have revealed that the reduction results from the conversion of glycosides and esters. The levels of functional components and antioxidant activities are elevated, and the free form enhances bioavailability [19,20]. Furthermore, free phenol and 5-HMF have α-glucosidase inhibitory ability, which can slow carbohydrate digestion in the intestine and aid in maintaining stable postprandial blood sugar levels [19,21,22].

Tangerine peel, a dried mature citrus peel, is one traditional Chinese medicine used to treat metabolic syndromes such as dyspepsia and inflammatory respiratory diseases [23]. It contains flavonoid components such as flavanone glycosides and polymethoxyflavonoids (hesperidin and narirutin) with antioxidant, anticancer, and anti-inflammatory characteristics [2,4,24,25]. Polymethoxyflavonoids are a type of flavonoid found exclusively in citrus peels that have a chemical structure containing more than two methoxy groups. They generate aldehyde groups with reducing power during oxidation. They have higher physiological activities than flavanone glycosides [5]. However, their preparation require substantial time and, thus, personnel costs. This study applies aging fermentation technology to whole oranges to develop black oranges, improve their physicochemical properties and functionality, reduce preparation time, and promote an environmentally friendly green processing method for a recycling economy. In addition, using the whole fruit to adjust market imbalances caused by climate change and increase the use of orange byproducts provides advantages such as increased value, reduced costs, and enhanced market stability, aligning with environmental concerns, material sustainability, and the potential to enhance human health and well-being [26].

This study uses aging fermentation technology to develop black oranges and accelerate their fermentation process with various pre-treatments. The oranges were assessed in four groups: fresh (F), non-blanching (NB), blanching (CA), and hot air-assisted aging cycle (AA). The specific aging conditions were 55 °C and 75% relative humidity for six weeks. This study examined the oranges’ physicochemical properties (pH, soluble solids, reducing sugars, polyphenolic compounds, ascorbic acid, saccharides, browning degree, and 5-HMF) and functional characteristics (total polyphenols, total flavonoids, 2,2-diphenyl-1-picrylhydrazyl free radical-scavenging capacity, ferric ion-reducing antioxidant power, and α-glucosidase inhibitory ability). In addition, we measured the FTIR spectrum of oranges and speculated on the characteristic bonding changes during processing based on the bonding strength of functional groups. Principal component analysis (PCA) was used to assess the browning properties, antioxidant activities, and α-glucosidase inhibitory ability of whole oranges, which can be used as a reference for future green processing and functional food industry applications.

## 2. Materials and Methods

### 2.1. Materials and Chemicals

The study procured fresh oranges (*Citrus sinensis* (L.) Osbeck) from a fruit store in Taichung, Taiwan. Each orange had an average weight of roughly 185 ± 10 g. All chemicals used were of analytical quality and were manufactured by Sigma-Aldrich Co. (Burlington, MA, USA).

### 2.2. Preparation of Black Oranges

In this study, oranges were categorized into four distinct groups. Consult Table 1 for various processing conditions. The dryer (Model 3926TB, Excalibur Dehydrator Co., Sacramento, CA, USA) was used for hot air drying, and a constant temperature and humidity machine (Model BTH80/-20, Firstek Co., New Taipei, Taiwan) was used for aging fermentation. The fresh oranges (F) that underwent sorting and cleaning were designated as the experimental control group. The NB group underwent aging fermentation in a controlled environment with constant temperature and humidity for a duration of 6 weeks at 55 °C and 75% relative humidity. The CA group was subjected to blanching at a temperature of 95 ± 2 °C for a duration of 5 min, whereas the NB group did not undergo this treatment. The AA group utilizes a combination of hot air drying and aging fermentation cycles. Each cycle consists of one day of hot air drying followed by six days of aging fermentation, for a total duration of six cycles. The samples underwent freeze-drying, grinding, and sieving through a 60-mesh sieve. They were then stored at a temperature of −80 °C until they were ready for analysis.

### 2.3. Quality Characteristics of Black Oranges

#### 2.3.1. Appearance

The photos were captured and observed using a digital monocular (Model 450D, Canon Co., Tokyo, Japan) lens in conjunction with a microscope camera (Model E3ISPM 20000KPA, Sony Inc., Tokyo, Japan).

#### 2.3.2. Color Analysis

The lightness value (L*), red-green chromaticity (a*), and yellow-blue chromaticity (b*) of the black orange sample were quantified using a colorimeter (Model NE-4000, Nippon Denshku Industries Co., Tokyo, Japan) and calibrated with a standard whiteboard (X = 92.81, Y = 94.83, Z = 111.71). Furthermore, the color difference value (ΔE) was calculated using Equation (1).

(1) $$\mathrm{Color}\;\mathrm{difference}\;(\Delta E)=\sqrt{{\left(L_{1}^{*}-\;L_{0}^{*}\right)}^{2}+{\left(a_{1}^{*}-a_{0}^{*}\right)}^{2}\;+(b_{1}^{*}-b_{0}^{*}{)}^{2}}$$

#### 2.3.3. Water Activity (Aw)

The water activity of black orange samples was determined using a water activity analyzer (Model Aqualab 3TE, Meter Group Inc., Washington, DC, USA) at a temperature of 25 °C.

#### 2.3.4. Total Weight and Weight Loss Ratio

The overall weight difference of the orange sample was measured and documented, then the rate of weight loss was computed using Equation (2).

(2) $$\mathrm{Weight}\;\mathrm{loss}\;\mathrm{ratio}\;(\%)=\frac{(W_{1}\;-\;W_{2})}{W_{1}}\times 100$$

where W_1_ is the original weight, and W_2_ is the weight of different aging treatments.

#### 2.3.5. Scanning Electron Microscope (SEM)

The morphology of black orange peel was examined using a field emission scanning electron microscope (FESEM) (Model JSM-7800F Prime, JEOL Ltd., Tokyo, Japan). The specimen was positioned on an aluminum rod with a platinum layer coated with platinum-palladium (Model JEC-3000FC, JEOL Ltd., Tokyo, Japan) and subsequently examined at an accelerating voltage of 3 kV.

#### 2.3.6. Fourier-Transform Infrared Spectroscopy (FTIR)

The black orange samples were examined using a Fourier-transform infrared spectrometer (FTIR) (Model Nicolet 6700, Thermo Fisher Scientific Co., Waltham, MA, USA) and an MCT IR detector (Thermo Fisher Scientific Co., Waltham, MA, USA), with a scanning range of 650 to 4000 cm^−1^.

### 2.4. Physical Characteristics of Black Oranges

A total of 5 g of dry sample powder was combined with 100 mL of deionized water and subjected to ultrasonic extraction for a duration of 60 min using an ultrasonic oscillator. The liquid portion was collected and passed through a 0.22 μm PTFE filter (Waters Co., Milford, MA, USA). The sample extract was stored at a temperature of −20 °C until it could be analyzed later.

#### 2.4.1. Browning Degree (A_420_)

Following the method by Nam et al. with modifications [20], the black orange extract was diluted by a factor of 2 with deionized water, and its absorbance was measured at a wavelength of 420 nm using a microplate reader (Model SPECTROstar Nano, BMG Labtech Co., Ortenberg, Germany). The sample’s absorbance value was measured, with deionized water serving as the blank control.

#### 2.4.2. pH

The pH of the black orange extract was determined using a pH meter (Model SP-2300, Suntex Instruments Co., New Taipei, Taiwan).

#### 2.4.3. Total Soluble Solids (TSS)

The soluble solid content (°Brix) of the black orange extract was determined using a portable refractometer (Model MASTER-M, ATOGO Co., Tokyo, Japan).

### 2.5. Chemical Composition Analysis of Black Oranges

The dry sample powder was combined with an 80% ethanol extract at a ratio of 1:10 (*w*/*v*), and the mixture was thoroughly mixed. The mixture was then subjected to ultrasonic oscillation for 60 min. After that, it was centrifuged at 6000 RCF for 10 min. The supernatant was then filtered through a 0.22 μm PTFE filter (Waters Co., Milford, MA, USA). The sample extract was stored at a temperature of −20 °C for further analysis.

#### 2.5.1. Total Reducing Sugars (TRS) and Sugar Analysis

The extract prepared in Section 2.5 was used for analysis. The total reducing sugar determination method by Lanthong et al. was followed and modified [27]. The high-performance liquid chromatography with a refractive index detector (HPLC-RI) is composed of a pump (Model Chromaster 5110, Hitachi Co., Tokyo, Japan) using a Shodex DC-613 column (150 mm, 6 mm i.d., 6.0 μm) (Resonac America Inc., New York, USA) and a refractive index detector (Model Chromaster 5450, Hitachi Co., Tokyo, Japan). A mobile phase consisting of 100% acetonitrile and 1.5 mM NaOH was utilized, and the analysis performed for a duration of 20 min. The temperature of the column was kept at 70 °C, the rate of flow was 1.5 mL min^−1^, and the volume of injection was 10 µL. The target compound is determined by comparing the retention time (min) of the standard. The amount of the target compound, measured in dry weight (mg g^−1^), is quantified using the calibration curve of the standard.

#### 2.5.2. Polyphenols Analysis

The method by Zhang et al. was followed and modified [28]. The extract prepared in Section 2.5 was used for analysis. The high-performance liquid chromatography with photo diode array detector (HPLC-DAD) is composed of a pump (Model Chromaster 5110, Hitachi Co., Tokyo, Japan) using a Mightysil RP-18GP column (250 mm, 4.6 mm i.d., 5.0 μm) (Kanto Co., Tokyo, Japan) and a photo diode array detector (Model L-2450, Hitachi Co., Tokyo, Japan). The mobile phase consists of 0.1% HCOOH (A) and 100% ACN (B), and the separation is performed using the following washing gradient: 0–5 min: 95% A, 5–20 min: 63% A, 20–25 min: 50% A, 25–30 min: 20% A, 30–35 min: 0% A (100% B), 35–40 min: 63 A, 40–45 min: 95% A. The rate of flow was 0.8 mL min^−1^, and the volume injected was 10 µL. The DAD detection wavelengths were configured at 280 nm and 330 nm, respectively, for the analysis of hesperidin and nobiletin. The target compound is determined by comparing the retention time (min) of the standard. The amount of the target compound, measured in dry weight (mg g^−1^), is quantified using the calibration curve of the standard.

#### 2.5.3. Ascorbic Acid Analysis

Following and modifying the method by Scherer et al. [29], the extract prepared in Section 2.5 was used for analysis. HPLC-DAD is composed of a pump (Model Chromaster 5110, Hitachi Co., Tokyo, Japan) using a Mightysil RP-18GP Aqua column (250 mm, 4.6 mm i.d., 5.0 μm) (Kanto Co., Tokyo, Japan) and a photo diode array detector (Model L-2450, Hitachi Co., Tokyo, Japan). The mobile phase was produced using a gradient of double-distilled water and 0.02 M ammonium dihydrogen phosphate buffer (pH 2.2 ± 0.2). The analysis of the mobile phase lasted for 20 min. The rate of flow was 1.0 mL min^−1^, and the volume injected was 10 µL. The wavelength for detecting DAD was configured at 243 nm. The target compound is determined by comparing the retention time (min) of the standard. The amount of the target compound, measured in dry weight (mg g^−1^), is quantified using the calibration curve of the standard.

#### 2.5.4. 5-Hydroxymethylfurfural Analysis

Following and modifying the method by Lee et al. [30], the extract prepared in Section 2.5 was used for analysis. The HPLC-DAD parameter was the same as in Section 2.5.2. The mobile phase consisted of a mixture of deionized water and acetonitrile in a ratio of 88:12 (*v*/*v*). The gradient of the mobile phase remained constant during the analysis. The analysis was conducted for a duration of 20 min. The rate of flow was 1.0 mL min^−1^, and the volume injected was 10 µL. The wavelength for detecting DAD was configured at 284 nm. The target compound is determined by comparing the retention time (min) of the standard. The amount of the target compound, measured in dry weight (mg g^−1^), is quantified using the calibration curve of the standard.

### 2.6. Functionality and Antioxidant Activity of Black Oranges

One gram of dry sample powder was thoroughly mixed with an 80% ethanol extract in a ratio of 1:50 (*w*/*v*). The mixture was subjected to ultrasonic oscillation for 60 min and then centrifuged at 6000 RCF for 10 min. The supernatant was then filtered through a 0.22 μm PTFE filter (Waters Co., Milford, MA, USA). The sample extract was stored at a temperature of −20 °C for further analysis.

#### 2.6.1. Total Phenol Content (TPC) Determination

Following and modifying the method by Magro and de Castro [31], 70 μL of Folin–Ciocalteu reagent was added to 70 μL of the extraction solution made in Section 2.6. The solution was thoroughly shaken and mixed, then allowed to sit at room temperature for 3 min. Subsequently, 35 μL of 10% a Na_2_CO_3_ solution was added, mixed, and stored in a dark environment for a duration of 30 min. The absorbance at a wavelength of 735 nm was determined using a microplate reader (Model SPECTROstar Nano, BMG Labtech Co., Ortenberg, Germany). Gallic acid was used as the standard and quantified to dried weight (mg g^−1^) by calibration curves.

#### 2.6.2. Total Flavonoid Content (TFC) Determination

Following and modifying the method by Fakhri et al. [32], 10 μL of the extraction solution prepared in Section 2.6 was added to 60 μL of distilled water and 30 μL of a 5% NaNO_2_ solution. The mixture was shaken thoroughly to ensure even distribution and stored in a dark environment for 6 min. Subsequently, 25 μL of a 2.5% AlCl_3_ solution, 25 μL of a 2% NaOH solution, and 50 μL of distilled water were sequentially added. The mixture was then mixed and left to react at room temperature in a dark environment for 15 min. The absorbance at a wavelength of 510 nm was determined using a microplate reader (Model SPECTROstar Nano, BMG Labtech Co., Ortenberg, Germany). Quercetin was used as the standard and quantified to dried weight (mg g^−1^) by calibration curves.

#### 2.6.3. 2,2-Diphenyl-1-picrylhydrazyl (DPPH) Radical-Scavenging Capacity

Following and modifying the method by Chen et al. [33], 10 μL of the extraction solution prepared in Section 2.6 was added to 40 μL of 100 mM Tris-HCl buffer solution (pH 7.4), then 75 μL of 0.5 mM DPPH solution was mixed and reacted in a dark environment for 30 min. The absorbance at a wavelength of 517 nm was determined using a microplate reader (Model SPECTROstar Nano, BMG Labtech Co., Ortenberg, Germany). Trolox was used as the standard and quantified to dried weight (mg g^−1^) by calibration curves.

#### 2.6.4. Ferric-Reducing Antioxidant Power (FRAP)

Following and modifying the method by Xu et al. [34], 20 μL of the extraction solution prepared in Section 2.6 was added to 150 μL of FRAP reagent and stored in a dark environment for 10 min at 37 °C. The absorbance at a wavelength of 593 nm was determined using a microplate reader (Model SPECTROstar Nano, BMG Labtech Co., Ortenberg, Germany). Trolox was used as the standard and quantified to dried weight (μM g^−1^) by calibration curves.

### 2.7. In Vitro α-Glucosidase Inhibition Assay

Following and modifying the method by Hsieh et al. [19], 50 μL of the extraction solution prepared in Section 2.6 was added to 100 μL of α-glucosidase solution and reacted at 25 °C for 10 min. Then, 50 μL of a 5 mM p-NPG solution was added and reacted at 25 °C for 5 min. The absorbance at a wavelength of 400 nm was determined using a microplate reader (Model SPECTROstar Nano, BMG Labtech Co., Ortenberg, Germany). A total of 70 µg mL^−1^ acarbose was used as the positive control to convert the inhibitory capacity of the black orange extract. The background group was composed of 50 μL of the black orange extract, 100 μL of PBS solution, and 50 μL of p-NPG. The inhibitory capacity of the samples was calculated using Equation (3).

(3) $$\mathrm{Inhibitory}\;\mathrm{capacity}\;(\%)=\frac{\left(A_{\mathrm{sample}}-A_{\mathrm{background}}\right)}{\left(A_{\mathrm{acarbose}}-A_{\mathrm{background}}\right)}\times 100$$

### 2.8. Statistical Analysis

All results were shown as mean ± standard deviation (*n* = 3). Data were collected through one-way analysis of variation (ANOVA) using SPSS version 19.0 software (IBM Co., Armonk, NY, USA). Significance tests were determined by Duncan’s Multiple Range Test (DMRT), with *p* < 0.05 considered to be significantly different. Principal Component Analysis (PCA) and Agglomerative Hierarchical Clustering (AHC) were conducted using XLSTAT (Addinsoft Co., New York, NY, USA) software.

## 3. Results and Discussion

### 3.1. Appearance and Color of Black Oranges

**Figure 1:**
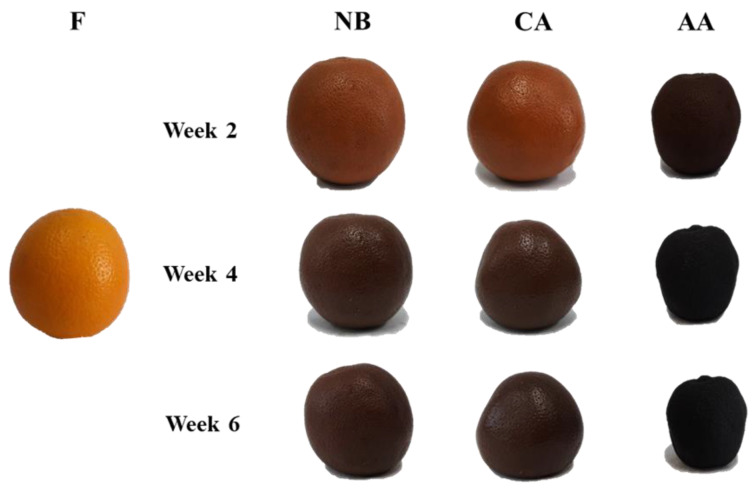
Appearance of black oranges under different aging fermentation processes. F, NB, CA, AA represent fresh, non-blanching and continuous aging, blanching and continuous aging, and hot air-assisted aging cycle, respectively.

Appearance and color greatly influence consumers’ willingness to accept and buy food products [15,18]. Figure 1 shows the visual transformations of the aging black oranges. The NB and CA groups resemble the F group due to the high temperature (55 °C) and relative humidity (75%) conditions, attributed to the high moisture content (75–90%) in the fresh fruit. In the CA group, blanching destroyed plant tissues, leading cells to swell and soften, resulting in a more pronounced contraction of the epidermal periphery after four weeks of aging [35]. A two-week hot air-assisted aging cycle treatment at 55 °C notably reduced internal moisture in the AA group, leading to visibly smaller fruit, firm texture, and an abrasive appearance.

Table 2 presents the color variations in the different groups of black oranges after six weeks of aging. Lightness (L*), redness (a*), and yellowness (b*) decreased significantly (*p* < 0.05): L* decreased from 60.37 to 14.39–26.85, a* decreased from 22.67 to 0.28–5.57, and b* decreased from 37.03 to 0.80–7.57. The color difference values (ΔE) for the NB, CA, and AA groups increased to 53.05, 52.89, and 62.11, respectively, which can be attributed to the browning response, consistent with the prior research by Zhu et al. [18]. The AA group had a greater matrix concentration, leading to an accelerated browning reaction rate, possibly due to the formation of Maillard reaction intermediate products [36]. The A_420_ value is commonly used to detect Maillard reaction intermediate products [15,17]. The absorbance of the black orange samples was quantified at a wavelength of 420 nm. The absorbance changed significantly after six weeks of aging, decreasing from 0.56 to 0.54 in the NB group, increasing to 0.61 in the CA group, and increasing to 3.44 in the AA group, respectively (*p* < 0.05; Table 2). Based on these results, black oranges primarily undergo non-enzymatic browning, and lowering the moisture through hot air drying might enhance their response rate [18,19].

### 3.2. Scanning Electron Microscope of Black Oranges

**Figure 2:**
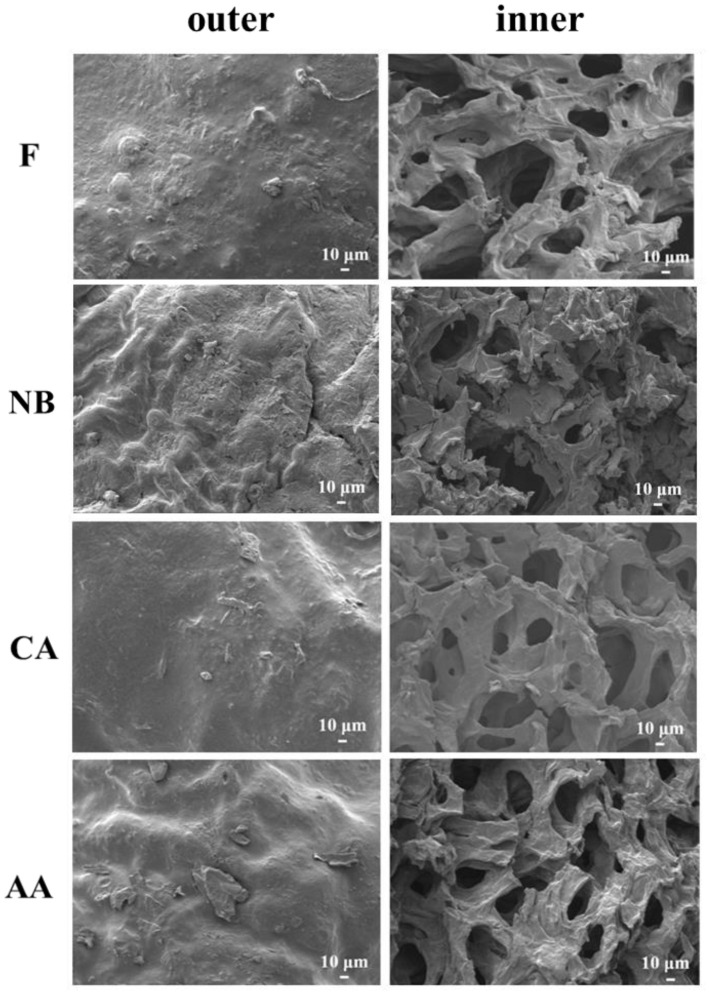
SEM images of black orange peel’s outer and inner structure under different aging fermentation processes for six weeks. SEM means scanning electron microscope. The SEM images were set as 300× magnification. F, NB, CA, AA represent fresh, non-blanching and continuous aging, blanching and continuous aging, and hot air-assisted aging cycle, respectively.

Scanning electron microscope is commonly used to examine the microstructure and alterations of samples for use as a point of reference in product development and improvement [37]. Figure 2 shows the microstructure of black oranges. We examined how various pre-treatments affect the structure of black orange peel by comparing its internal and surface features to regular orange peel [37]. After six weeks of aging, the NB group developed wrinkles compared to the F group due to water loss. In the CA group, the epidermis became smoother because of tissue structure destruction due to blanching treatment, softening of epidermal cells, and enhancing water retention capacity [35]. The creases in the AA group were less apparent due to volume contraction during hot air drying, which then stabilizes at a humidity equilibrium over the aging process.

Black oranges have higher porosity in their internal structure than fresh oranges. Previous studies indicate that hot air treatment causes water loss, destroys peel structure, and releases intracellular macromolecular phenolic chemicals as tiny free molecules [15,19]. In the AA group, the shrinkage and volume loss of the outer peel created smaller pores in the internal structure, resulting in an increased concentration of the browning reaction matrix and the release of intracellular bioactive components throughout the aging process. This observation demonstrates that small-molecule free phenolic acid production was accelerated in the AA group, whereas the NB and CA groups retained their original structure.

### 3.3. FTIR Spectrum of Black Oranges

**Figure 3:**
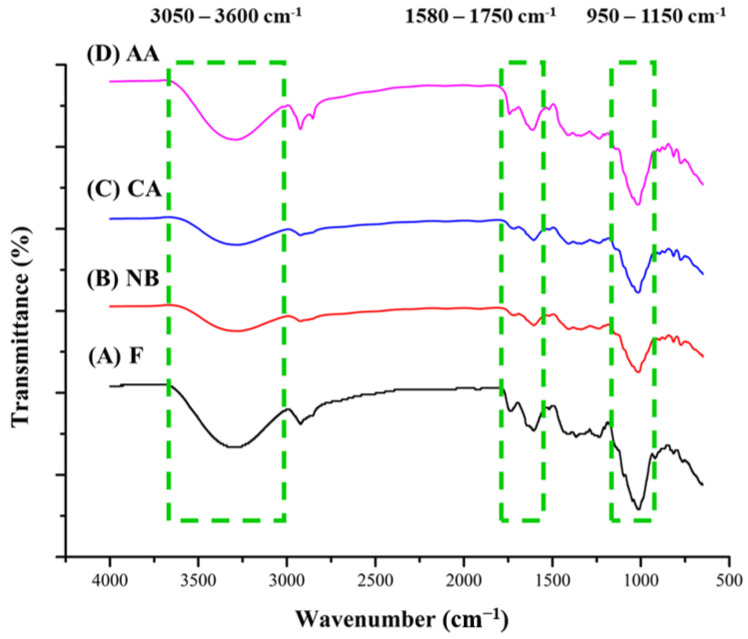
FTIR spectrum of black oranges under different aging fermentation processes. F, NB, CA, AA represent fresh, non-blanching and continuous aging, blanching and continuous aging, and hot air-assisted aging cycle, respectively.

FTIR can be used to measure the bonding strength of functional groups, enabling the detection of typical bonding changes during processing and facilitating hypotheses about the aging response mechanism. When fresh oranges were compared to black oranges aged for six weeks (Figure 3), a distinct band was observed in the wavelength range of 3050–3600 cm^−1^, indicating the stretching vibration of the OH group. This band is characteristic of phenolic chemicals and 5-HMF [38,39]. The bonding strength was higher in the AA group than in the F group due to the hot air-drying process, which accelerates the production of free polyphenols and enhances the browning reaction [15,19], consistent with the increase in total polyphenols, total flavonoids, and 5-HMF contents.

Aromatic compounds and carbonyl groups were detected in the wavelength range of 1580–1750 cm^−1^, indicating the stretching vibration of C=C and C=O groups [40]. Bond strengths were reduced in the NB, CA, and AA groups due to decreases in alkenes, aldehydes, ketones, and esters during the aging process. Decreasing the combinations of ester forms increases the presence of free phenols. The released compounds enhance the antioxidant activity of black oranges [18,19].

Carbohydrates were detected at the peak between 950 and 1150 cm^−1^, indicating the stretching vibration of the C-O-C group, which corresponds to the glycosidic bond [41]. This observation indicates that glycosidic bonds break down in black oranges during aging due to the browning reaction during thermal processing, decreasing the peak tensile vibration value. The AA group had a higher peak intensity than the NB and CA groups, suggesting a reduction in disaccharide content after six weeks of hot air-assisted aging cycle treatment. This decrease is consistent with the decrease in monosaccharide content resulting from glycosidic bond hydrolysis. It can be linked to the accelerated Maillard reaction rate, increased browning degree, and higher Maillard reaction product content in black oranges [13,14].

### 3.4. Physical Characteristics of Black Oranges

The water activity level during aging affects the speed of enzymatic and non-enzymatic browning reactions and can be an indication of these reactions [42]. Maintaining a water activity level between 0.65 and 0.85 is beneficial for non-enzymatic browning reactions. Therefore, measuring water activity is a crucial quality control measure when making aged products [42]. Table 3 presents the physical characteristics of black oranges. After six weeks of aging, water activity in the NB, CA, and AA groups decreased from 0.97 to 0.96, 0.96, and 0.54, respectively. The AA group experienced rapid water loss and structural breakdown, resulting in a rapid reduction in water activity throughout the hot air aging process and accelerated non-enzymatic browning reactions as the aging progressed (Aw 0.65–0.85). In contrast, the NB and CA groups maintained an intact peel structure that prevented internal water loss (Figure 1). The weights of black oranges decreased from 544.21 to 100.51–355.31 g after six weeks of aging due to water evaporation (*p* < 0.05; Table 3). We also assessed the weight loss ratio in black oranges. After six weeks of aging, the weight loss ratios for the NB, CA, and AA groups were 34.71, 36.00, and 81.53%, respectively. There were significant increases in the weight loss ratio and water activity change in each group (*p* < 0.05). The weight loss ratio is a metric that assesses a product’s production cost and yield, serving as a benchmark for future large-scale manufacturing.

Table 3 also presents the pH changes in black oranges during the aging process. The fresh oranges had a pH of 4.73. After six weeks of aging, the pH significantly decreased to 3.69–3.80 (*p* < 0.05), reflecting the breakdown of the orange’s structure and the browning process. Factors such as the release of organic acids due to plant cell wall rupturing and the browning response leading to the breakdown of carbohydrates into pyruvate or acetic acid are connected to the reduction in pH [18,20,43]. The soluble solids content of black oranges aged for six weeks was significantly higher than that of fresh oranges (*p* < 0.05; Table 3). Sugar contents in the NB, CA, and AA groups increased from 2.62 to 3.15, 3.10, and 3.14 °Brix, respectively, due to non-enzymatic browning as they aged [10,11]. When exposed to heat, polysaccharides are broken down into monosaccharides by hydrolysis of glycosidic bonds, leading to higher soluble solid content and enhanced sweetness. Furthermore, reducing sugar is a key indicator when evaluating the browning reaction. After four weeks of aging, the total reducing sugar content of the NB and CA groups increased significantly from 278.28 to 545.86 and 555.34 mg/g D.W., respectively, compared to the F group (*p* < 0.05; Table 3). In the AA group, the reducing sugar content increased to 524.04 mg/g D.W. after two weeks of aging and then decreased to 477.94 mg/g D.W. after six weeks [19,36]. The initial increase in reducing sugar content was caused by the thermal breakdown of polysaccharides in the oranges’ cell walls. During the later stage, prolonged hot air drying decreases moisture content, increases the matrix concentration of black oranges, and accelerates the Maillard reaction rate, leading to a reduction in reducing sugar content [36,44].

### 3.5. Chemical Composition of Black Orange

Table 4 presents the changes in the chemical composition of black oranges after aging. Hesperidin and nobiletin concentrations were 114.34 and 0.47 mg/g D.W., respectively, in fresh oranges, decreasing significantly to 40.10–49.65 and 0.28–0.43 mg/g D.W., respectively, after six weeks of aging (*p* < 0.05). Research has shown that under specific conditions, macromolecular polyphenolic compounds break down into free phenolic acids with increased antioxidant activity after prolonged exposure to high temperatures and high humidity [3,15,19]. Ascorbic acid, which has antioxidant, anti-inflammatory, and other biological characteristics, decreased with aging (*p* < 0.05). After six weeks of aging, the ascorbic acid levels in the NB, CA, and AA groups decreased to 0.47, 0.51, and 0.57 mg/g D.W., respectively (Table 4), consistent with other research suggesting pyrolysis occurs in acidic, high-temperature environments [1,12,45,46].

Table 4 also presents the changes in sugars found in black oranges after aging. After four weeks of aging, the sucrose content was significantly lower in the NB, CA, and AA groups than in the F group, decreasing from 96.96 to 4.68, 11.72, and 1.66 mg/g D.W., respectively (*p* < 0.05). The glucose content increased from 91.44 to 168.84 and 178.26 mg/g D.W. after six weeks of aging in the NB and CA groups, and the fructose content increased from 106.07 to 223.26 and 237.85 mg/g D.W., respectively. The disaccharides break down into glucose and fructose via glycosidic bond hydrolysis [10,11]. Glucose is also isomerized into fructose through the Maillard reaction. The levels of glucose and fructose in the AA group increased to 167.93 and 231.34 mg/g D.W. after two weeks of aging and then decreased to 110.92 and 169.61 mg/g D.W. after six weeks.

Heat treatment was associated with changes in sugar content. The increase in monosaccharide levels during the initial phase of aging was caused by the breakdown of polysaccharides. The decrease in monosaccharide levels during the later phase might be associated with the Maillard process generating 5-HMF in acidic environments. During this phase, the consumption rate of monosaccharides is greater than their production rate [20,36,47]. This result shows that water activity in the AA group falls within a range associated with higher non-enzymatic browning reaction rates, indicating a greater consumption rate of monosaccharides than in the NB and CA groups. Previous studies have demonstrated that 5-HMF has antioxidant, anti-inflammatory, and other beneficial characteristics [11,48]. After six weeks of aging, the 5-HMF content in the AA group increased to 0.09 mg/g D.W. (Table 4) [19,36].

### 3.6. Functionality and Antioxidant Activity of Black Oranges

**Figure 4:**
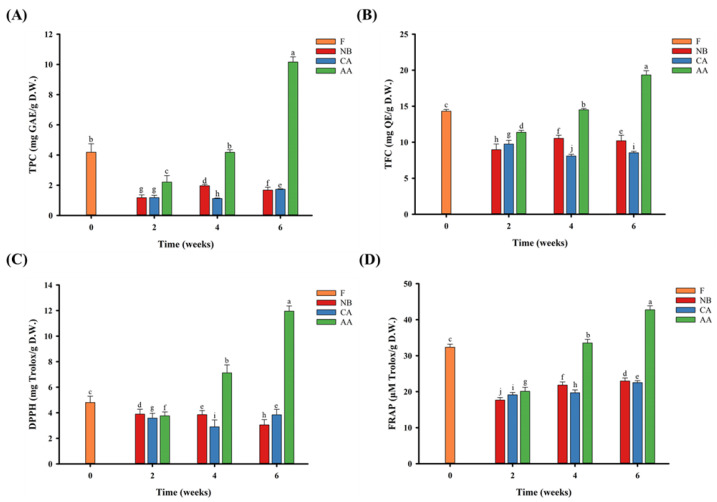
Analysis of functionality and antioxidant activity of black oranges under different aging fermentation processes. (**A**) Total phenol content. (**B**) Total flavonoid content. (**C**) 2,2-Diphenyl-1-picrylhydrazyl (DPPH) radical-scavenging capacity. (**D**) Ferric-reducing antioxidant power. “a–j” means different letters in the figure have significant differences (*p* < 0.05). F, NB, CA, AA represent fresh, non-blanching and continuous aging, blanching and continuous aging, and hot air-assisted aging cycle, respectively.

Fresh oranges contain ascorbic acid and flavonoids, which function as antioxidants and benefit human health [1,3,5]. The antioxidant activities of black oranges were assessed by analyzing their total polyphenols, total flavonoids, DPPH free radical-scavenging capacity, and ferric ion-reducing antioxidant power (FRAP) (Figure 4). Figure 4A,B shows that fresh oranges contain 4.18 mg GAE/g D.W. of total polyphenols and 14.31 mg QE/g D.W. of total flavonoids. After six weeks of aging, the total polyphenol and flavonoid contents were 1.68–10.16 mg GAE/g D.W. and 8.54–19.35 mg QE/g D.W., respectively. Their contents increased significantly in the AA group (*p* < 0.05). The increase in quercetin content among total flavonoids was associated with the conversion of rutin to quercetin due to the heat treatment, leading to decreased aglycone-form binding [49]. Similarly, Choi et al. [15] found that total polyphenol and flavonoid contents were higher in black garlic than in raw garlic. Heat treatment can liberate polyphenols, transforming them from large into tiny molecules, enhancing the concentration of free polyphenols, decreasing the formation of ester and glycoside compounds, and ultimately increasing the levels of total polyphenols and flavonoids [15,19]. In Figure 1, the decreased volume of black oranges in the AA group, influenced by the matrix effect, accelerated the production of small-molecule free phenolic acids, leading to a notable increase in antioxidant levels, consistent with the prior research by Mohdaly et al. [50].

Free radicals are produced during metabolic processes in the body and consist of molecules, atoms, or ions with unpaired electrons. Due to their high reactivity and instability, free radicals induce oxidative interactions with cells in the body, resulting in damage to normal cells and impaired function. The excessive accumulation of free radicals can cause cell damage, oxidative stress, and related disorders such as diabetes, cancer, and cardiovascular disease [51]. The antioxidant activities of DPPH and FRAP are assessed by counteracting free radicals via electron transfer. Robust antioxidant activity suggests that antioxidant compounds can protect cells from oxidative harm and prevent illness [13,14].

Figure 4C,D shows the results of the antioxidant activity assessments of black oranges. The changes in DPPH free radical-scavenging capacity and ferric ion-reducing antioxidant power (FRAP) are comparable and correlate positively with total polyphenol and total flavonoid contents. The DPPH and FRAP activities were 4.80 mg Trolox/g D.W. and 32.37 μM Trolox/g D.W., respectively, in the F group. After six weeks of aging, they decreased to 3.05–3.84 mg Trolox/g D.W. and 22.52–22.97 μM Trolox/g D.W. in the NB and CA groups, whereas they increased to 11.95 mg Trolox/g D.W. and 42.75 μM Trolox/g D.W., respectively, in the AA group (*p* < 0.05). The antioxidant activities decreased in the NB and CA groups due to the faster oxidation than breakdown of phenolic compounds and the decrease in total polyphenols and total flavonoid contents. The antioxidant activity increased in the AA group due to the significant increases in total polyphenol and total flavonoid contents (*p* < 0.05). According to Zhu et al. [18], the increase in antioxidant activity results from the creation of Maillard reaction products during heat processing and the release of free polyphenols after the thermal treatment of polyphenols. Research indicates that free phenolic acids exhibit greater antioxidant potential. Antioxidant activity is enhanced by altering the number of hydroxyl groups on the benzene ring of polyphenols, and the ability to scavenge free radicals is improved by electron transfer [14,52].

### 3.7. In Vitro α-Glucosidase Inhibitory Potential of Black Oranges

**Figure 5:**
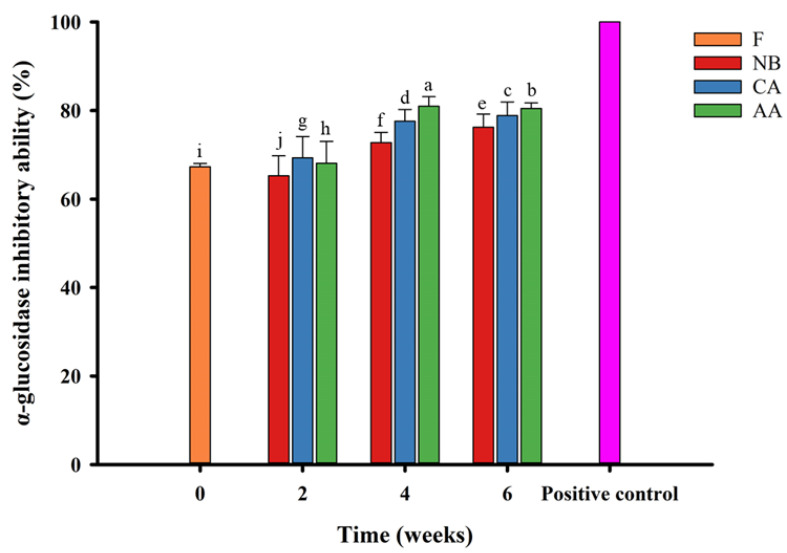
Inhibition of in vitro α-glucosidase ability of black oranges under different aging fermentation processes. Positive control means 100% control group with 70 µg/mL of acarbose. “a–j” means different letters in the figure have significant differences (p < 0.05). F, NB, CA, AA represent fresh, non-blanching and continuous aging, blanching and continuous aging, and hot air-assisted aging cycle, respectively.

α-glucosidase is present in the brush border of the small intestine in humans. Its function is to break down carbohydrates into glucose, increasing blood sugar levels after eating [21,22,53]. Therefore, many recent health food studies have focused on managing postprandial blood sugar levels. Fresh oranges were found to inhibit α-glucosidase activity by approximately 67.31% (Figure 5). After aging for six weeks, α-glucosidase inhibitory abilities had increased significantly in the NB, CA, and AA groups of black oranges to 76.26, 78.86, and 80.48%, respectively, suggesting that the aging process enhanced the α-glucosidase inhibitory ability of black oranges (*p* < 0.05). According to Hsieh et al. [19], the increase in α-glucosidase inhibitory ability after treatment in a high temperature and high humidity environment is related to an increase in free polyphenol and 5-HMF contents. The AA group exhibited the greatest inhibitory ability among groups. The free polyphenol release and 5-HMF content increased, showing promise for creating healthcare products with antioxidant activities and the ability to regulate postprandial blood sugar levels.

### 3.8. Principal Component Analysis of Black Oranges

**Figure 6:**
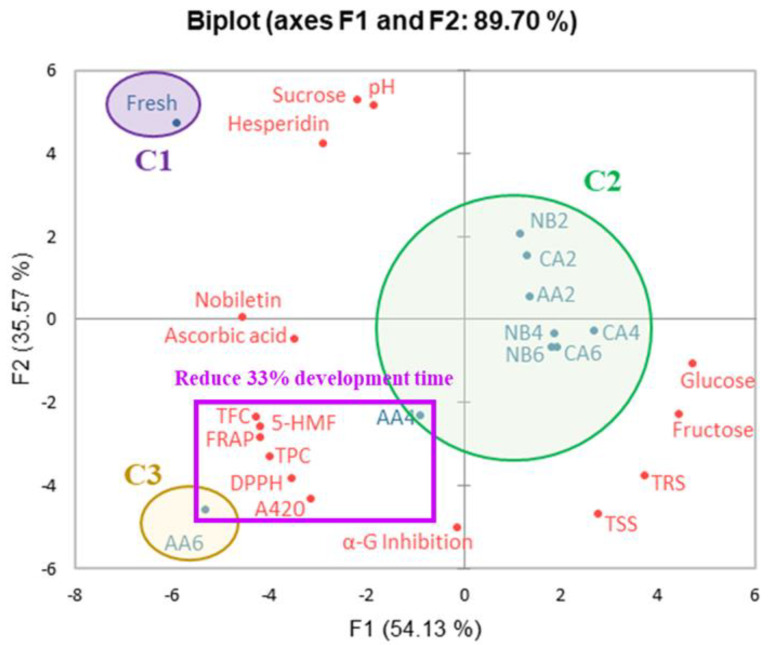
Principal component analysis of black oranges under different aging fermentation processes. NB2, NB4, NB6, CA2, CA4, CA6, AA2, AA4, AA6 represent non-blanching and continuous aging for 2, 4, and 6 weeks, blanching and continuous aging for 2, 4, and 6 weeks, and hot air-assisted aging cycle for 2, 4, and 6 weeks, respectively.

PCA is a technique used to decrease the dimensionality of a dataset. It uses the smallest number of dimensions to explain the dataset and diagrams for simplification and visualization to determine the association between clusters based on significant differences (*p* < 0.05) in agglomerative hierarchical clustering (AHC) [54]. Figure 6 shows that principal components one (F1) and two (F2) accounted for 54.13% and 35.57% of the variance in the data, respectively. Overall, the PCA accounted for 89.70% of the total variance in the dataset. The fresh oranges were included in cluster one (C1), located in the top left corner of the plot. They have significant levels of sucrose and hesperidin and a specific pH, which contributes to their sweet and sour taste and antioxidant activities.

NB2, NB4, and NB6 and CA2, CA4, and CA6 represent non-blanching and blanching aging processes lasting two, four, and six weeks, respectively, and AA2 and AA4 represent hot air-assisted aging cycles lasting two and four weeks. These processes were classified into cluster two (C2), located on the right side of the plot. They indicate that the glycosidic bond in sucrose is hydrolyzed, increasing glucose, fructose, and total reducing sugar contents, thereby enhancing the sweetness of black oranges. AA6 represents a hot air-assisted aging cycle that lasts for six weeks. It was categorized into cluster three (C3), located in the lower-left corner of the plot. The process notably enhanced the total polyphenol and flavonoid contents, browning degree, 5-HMF, DPPH, and FRAP.

In addition, nobiletin and ascorbic acid were categorized into clusters one (C1) and three (C3). After a six-week hot air-assisted aging cycle treatment, the nobiletin and ascorbic acid contents of black oranges were similar to those of fresh oranges, potentially due to the liberation of free polyphenols after heat treatment [15,19]. Clusters two (C2) and three (C3) show similar levels of soluble solid content and α-glucosidase inhibitory ability, suggesting that the higher monosaccharide content and robust α-glucosidase inhibitory ability of all groups of black oranges after aging treatment could be associated with the Maillard reaction during heat treatment and the formation of its intermediate product, 5-HMF [19,21]. The statistical data indicate that the AA group is optimal for developing black oranges. Furthermore, there was no significant disparity in the statistical outcomes among AA4, NB6, and CA6, suggesting that using AA to prepare black oranges could reduce the time by two weeks, enhance production efficiency, and lower production expenses. With an increase in aging time, the water activity of the AA group experiences a considerable decline, dropping from 0.95 to 0.54. Water activity regulation governs the rates of enzymatic and non-enzymatic reactions. Hence, AA treatment can significantly decrease the amount of time required for preparing black oranges [10,42]. 

### 3.9. Storage test of Black Oranges

Light, temperature, and time are important factors in the degradation of bioactive components, such as flavonoids, which can be easily isomerized or degraded by light to change a product’s color and reduce flavonoid content [33,55,56]. In this research, the black oranges were placed at 4, 25, and 55 °C with and without light storage tests, and the tests were carried out as shown in Table 5 to evaluate the water activity, color change, and stability of the bioactive ingredient. After sixty days of storage, the water activity of the NB and AA groups in the light and dark environments decreased to 0.72–0.86 and 0.27–0.42, respectively, which can be attributed to internal water loss. Compared with the dark environment, the lightness (L*), redness (a*), and yellowness (b*) in the light environment samples changed significantly during storage. Following the increasing storage time, L* decreased from 23.75–29.55 to 15.75–24.93, a* decreased from 5.53–8.22 to 0.32–6.90, and b* decreased from 5.03–7.94 to 0.57–6.59. The obvious decrease in the 55 °C NB group is related to high temperature and appropriate moisture content, which can accelerate the Maillard reaction [15,19].

In addition, the color difference values (ΔE) for different storage groups increased to 2.04–15.10, which can be attributed to the browning response, consistent with the prior research by Fu et al. [23]. Table 5 shows the concentration of hesperidin and nobiletin stored in light and dark environments. Exposure to light had an obvious effect on the black oranges. After sixty days of storage, hesperidin and nobiletin concentrations in the light environment decreased to 27.24–28.27 and 0.20–0.32, respectively. Moreover, the concentrations of hesperidin and nobiletin decreased to 28.15–29.51 and 0.24–0.36 in the dark environment, respectively. Therefore, AA group black orange can retain more flavonoid compounds when stored at 4 and 25 °C, with less color change and improved storage stability.

## 4. Conclusions

This study examined the browning properties, antioxidant activities, and α-glucosidase inhibitory ability of oranges after aging fermentation. It observed an increase in the color difference values (ΔE), weight loss ratios, and soluble solids contents of black oranges after six weeks of aging fermentation. The sucrose content decreased, whereas the glucose and fructose contents increased, due to glycosidic bond hydrolysis. The hot air-assisted aging cycle (AA) treatment accelerated the formation of black oranges and decreased the development time by 33%. Furthermore, AA significantly enhanced the degree of browning; 5-HMF, total polyphenol, and total flavonoid contents; DPPH free radical-scavenging capacity; and ferric ion-reducing antioxidant power (*p* < 0.05). The α-glucosidase inhibitory ability increased from 67.31 to 80.48%, showing promise in managing postprandial blood sugar levels. This study integrated green processing technologies to offer insights into future developments in whole-fruit aging fermentation products and the control of healthcare functions.

## Figures and Tables

**Table 1:** Processing parameters of different aging fermentation on black oranges.

Group	Blanching	Hot Air Drying	Aging
Condition	95 ± 2 °C, 5 min	55 °C, 1 day	55 °C, 75% RH, 6 weeks
F	–	–	–
NB	–	–	●
CA	●	–	●
AA	–	●	●

RH represents relative humidity. F, NB, CA, AA represent fresh, non-blanching and continuous aging, blanching and continuous aging, and hot air-assisted aging cycle, respectively. “●”means the orange sample group prepared included that treatment. “–” means the orange sample group prepared was not applicable to that treatment.

**Table 2:** Color changes of black oranges under different aging fermentation processes.

Week	Group	L*	a*	b*	ΔE	A_420_
0	F	60.37 ± 0.01 ^a^	22.67 ± 0.02 ^a^	37.03 ± 0.01 ^a^	–	0.56 ± 0.01 ^d^
2	NB	38.85 ± 0.01 ^b^	11.63 ± 0.05 ^b^	11.60 ± 0.02 ^c^	36.46 ± 0.02 ^h^	0.53 ± 0.01 ^e^
	CA	36.65 ± 0.01 ^c^	6.30 ± 0.03 ^d^	14.30 ± 0.01 ^b^	29.79 ± 0.02 ^i^	0.43 ± 0.00 ^g^
	AA	23.68 ± 0.02 ^g^	4.98 ± 0.04 ^f^	5.62 ± 0.01 ^g^	51.37 ± 0.04 ^e^	0.38 ± 0.00 ^h^
4	NB	30.79 ± 0.03 ^d^	9.36 ± 0.04 ^c^	11.35 ± 0.02 ^d^	46.89 ± 0.01 ^f^	0.45 ± 0.01 ^f^
	CA	25.78 ± 0.01 ^f^	4.90 ± 0.00 ^g^	9.35 ± 0.00 ^e^	40.90 ± 0.01 ^g^	0.38 ± 0.01 ^h^
	AA	16.58 ± 0.00 ^h^	0.75 ± 0.06 ^i^	0.82 ± 0.02 ^i^	60.83 ± 0.03 ^b^	1.39 ± 0.03 ^b^
6	NB	26.85 ± 0.05 ^e^	5.57 ± 0.01 ^e^	7.57 ± 0.01 ^f^	53.05 ± 0.02 ^c^	0.54 ± 0.02 ^e^
	CA	14.39 ± 0.01 ^j^	3.22 ± 0.01 ^h^	4.38 ± 0.02 ^h^	52.89 ± 0.03 ^d^	0.61 ± 0.01 ^c^
	AA	15.01 ± 0.02 ^i^	0.28 ± 0.00 ^j^	0.80 ± 0.02 ^j^	62.11 ± 0.01 ^a^	3.44 ± 0.07 ^a^

Each value was expressed as mean ± standard deviation (*n* = 3). “a–j” means different letters in the same column have significant differences (*p* < 0.05). “–” means ΔE was not applicable to fresh samples. F, NB, CA, AA represent fresh, non-blanching and continuous aging, blanching and continuous aging, and hot air-assisted aging cycle, respectively.

**Table 3:** Physical characteristics of black oranges under different aging fermentation processes.

Week	Group	Aw	Total Weight (g)	Weight Loss Ratio (%)	pH	TSS (°Brix)	TRS (mg/g D.W.)
0	F	0.97 ± 0.01 ^a^	544.21 ± 0.00 ^a^	0.00 ± 0.00 ^j^	4.73 ± 0.01 ^a^	2.62 ± 0.00 ^g^	278.28 ± 0.82 ^j^
2	NB	0.96 ± 0.00 ^ab^	415.14 ± 0.00 ^d^	23.72 ± 0.00 ^g^	4.08 ± 0.00 ^b^	2.99 ± 0.01 ^f^	473.95 ± 3.25 ^h^
	CA	0.96 ± 0.01 ^ab^	450.75 ± 0.00 ^b^	17.17 ± 0.00 ^i^	4.07 ± 0.00 ^b^	3.11 ± 0.00 ^d^	460.74 ± 2.61 ^i^
	AA	0.95 ± 0.00 ^bc^	267.15 ± 0.00 ^h^	50.91 ± 0.00 ^c^	3.88 ± 0.00 ^c^	3.05 ± 0.00 ^e^	524.04 ± 2.45 ^e^
4	NB	0.95 ± 0.01 ^bc^	409.34 ± 0.00 ^e^	24.78 ± 0.00 ^f^	3.85 ± 0.00 ^d^	3.14 ± 0.01 ^c^	545.86 ± 4.84 ^d^
	CA	0.94 ± 0.01 ^c^	442.54 ± 0.01 ^c^	18.68 ± 0.01 ^h^	3.89 ± 0.00 ^c^	3.21 ± 0.01 ^b^	555.34 ± 5.63 ^b^
	AA	0.71 ± 0.00 ^d^	114.61 ± 0.01 ^i^	78.94 ± 0.01 ^b^	3.74 ± 0.00 ^g^	3.24 ± 0.01 ^a^	517.58 ± 4.47 ^f^
6	NB	0.96 ± 0.00 ^ab^	355.31 ± 0.01 ^f^	34.71 ± 0.02 ^e^	3.78 ± 0.01 ^f^	3.15 ± 0.00 ^c^	563.47 ± 4.04 ^a^
	CA	0.96 ±0.00 ^ab^	348.27 ± 0.00 ^g^	36.00 ± 0.01 ^d^	3.80 ± 0.01 ^e^	3.10 ± 0.01 ^d^	547.45 ± 3.73 ^c^
	AA	0.54 ± 0.01 ^e^	100.51 ± 0.01 ^j^	81.53 ± 0.01 ^a^	3.69 ± 0.02 ^h^	3.14 ± 0.01 ^c^	477.94 ± 3.41 ^g^

Each value was expressed as mean ± standard deviation (*n* = 3). “a–j” means different letters in the same column have significant difference (*p* < 0.05). Aw means water activity, TSS means total soluble solids, TRS means total reducing sugars. F, NB, CA, AA represent fresh, non-blanching and continuous aging, blanching and continuous aging, and hot air-assisted aging cycle, respectively.

**Table 4:** Chemical compositions of black oranges under different aging fermentation processes.

Week	Group	Hesperidin (mg/g D.W.)	Nobiletin (mg/g D.W.)	Ascorbic Acid (mg/g D.W.)	Sucrose (mg/g D.W.)	Glucose (mg/g D.W.)	Fructose (mg/g D.W.)	5-HMF (mg/g D.W.)
0	F	114.34 ± 0.13 ^a^	0.47 ± 0.02 ^a^	0.66 ± 0.01 ^a^	96.96 ± 0.14 ^a^	91.44 ± 0.10 ^j^	106.07 ± 0.23 ^j^	–
2	NB	47.53 ± 0.13 ^f^	0.30 ± 0.00 ^e^	0.24 ± 0.02 ^h^	64.41 ± 0.11 ^b^	153.81 ± 0.14 ^g^	199.86 ± 0.14 ^h^	–
	CA	45.81 ± 0.08 ^g^	0.31 ± 0.01 ^e^	0.24 ± 0.00 ^h^	60.12 ± 0.07 ^c^	154.57 ± 0.09 ^f^	205.17 ± 0.09 ^f^	–
	AA	53.90 ± 0.15 ^b^	0.35 ± 0.02 ^d^	0.31 ± 0.00 ^g^	18.66 ± 0.10 ^d^	167.93 ± 0.12 ^e^	231.34 ± 0.17 ^c^	–
4	NB	43.16 ± 0.13 ^i^	0.31 ± 0.01 ^e^	0.39 ± 0.01 ^e^	4.68 ± 0.14 ^f^	177.97 ± 0.10 ^b^	225.68 ± 0.15 ^d^	–
	CA	47.75 ± 0.05 ^e^	0.26 ± 0.01 ^g^	0.35 ± 0.02 ^f^	11.72 ± 0.11 ^e^	176.51 ± 0.16 ^c^	235.90 ± 0.23 ^b^	–
	AA	51.06 ± 0.09 ^c^	0.41 ± 0.00 ^c^	0.39 ± 0.01 ^e^	1.66 ± 0.11 ^i^	149.94 ± 0.08 ^h^	202.52 ± 0.17 ^g^	0.02 ± 0.02 ^b^
6	NB	45.48 ± 0.09 ^h^	0.28 ± 0.00 ^f^	0.47 ± 0.10 ^d^	4.67 ± 0.10 ^f^	168.84 ± 0.10 ^d^	223.26 ± 0.22 ^e^	–
	CA	49.65 ± 0.11 ^d^	0.28 ± 0.01 ^f^	0.51 ± 0.02 ^c^	3.91 ± 0.16 ^g^	178.26 ± 0.13 ^a^	237.85 ± 0.19 ^a^	–
	AA	40.10 ±0.01 ^j^	0.43 ±0.00 ^b^	0.57 ± 0.07 ^b^	3.88 ± 0.09 ^h^	110.92 ± 0.14 ^i^	169.61 ± 0.24 ^i^	0.09 ± 0.01 ^a^

Each value was expressed as mean ± standard deviation (*n* = 3). “a–j” means different letters in the same column have significant differences (*p* < 0.05). “–” means not detected. F, NB, CA, AA represent fresh, non-blanching and continuous aging, blanching and continuous aging, and hot air-assisted aging cycle, respectively.

**Table 5:** Changes in black oranges during storage at 4, 25, 55 °C for 60 days.

Day	Environment	Group		Aw	L*	a*	b*	ΔE	Hesperidin (mg/g D.W.)	Nobiletin (mg/g D.W.)
0	Control	NB	25 °C	0.95 ± 0.01 ^a^	29.55 ± 0.02 ^a^	8.22 ± 0.08 ^a^	7.94 ± 0.06 ^a^	–	45.12 ± 0.13 ^a^	0.25 ± 0.02 ^h^
		AA	25 °C	0.45 ± 0.01 ^e^	23.75 ± 0.01 ^f^	5.53 ± 0.04 ^e^	5.03 ± 0.03 ^e^	–	40.21 ± 0.13 ^b^	0.41 ± 0.01 ^a^
30	Light	NB	4 °C	0.89 ± 0.00 ^b^	23.70 ± 0.01 ^f^	6.51 ± 0.05 ^c^	5.19 ± 0.02 ^e^	1.89 ± 0.03 ^k^	30.52 ± 0.09 ^f^	0.26 ± 0.00 ^g^
			25 °C	0.90 ± 0.01 ^b^	24.58 ± 0.02 ^e^	6.89 ± 0.03 ^b^	6.42 ± 0.01 ^c^	5.37 ± 0.01 ^c^	30.14 ± 0.13 ^f^	0.23 ± 0.02 ^i^
			55 °C	0.90 ± 0.00 ^b^	17.75 ± 0.01 ^k^	0.71± 0.01 ^l^	0.42 ± 0.03 ^k^	13.55 ± 0.02 ^b^	30.27 ± 0.13 ^f^	0.25 ± 0.02 ^h^
		AA	4 °C	0.42 ± 0.01 ^f^	20.36 ± 0.01 ^i^	4.09 ± 0.05 ^j^	3.22 ± 0.02 ^h^	2.77 ± 0.02 ^g^	31.20 ± 0.01 ^e^	0.39 ± 0.01 ^b^
			25 °C	0.41 ± 0.00 ^f^	22.07 ± 0.01 ^g^	5.32 ± 0.04 ^f^	4.45 ± 0.01 ^f^	1.79 ± 0.01 ^l^	31.75 ± 0.13 ^d^	0.37 ± 0.02 ^c^
			55 °C	0.36 ± 0.01 ^h^	23.75 ± 0.01 ^f^	5.02 ± 0.01 ^g^	4.72 ± 0.01 ^f^	2.97 ± 0.02 ^f^	30.56 ± 0.13 ^f^	0.36 ± 0.02 ^c^
	Dark	NB	4 °C	0.90 ± 0.00 ^b^	23.94 ± 0.01 ^e^	6.86 ± 0.04 ^b^	5.51 ± 0.02 ^d^	1.75 ± 0.03 ^l^	32.15 ± 0.09 ^d^	0.27 ± 0.01 ^f^
			25 °C	0.93 ± 0.01 ^b^	25.67 ± 0.02 ^b^	6.95 ± 0.03 ^b^	6.84 ± 0.01 ^b^	5.26 ± 0.01 ^c^	31.95 ± 0.13 ^d^	0.26 ± 0.03 ^g^
			55 °C	0.92 ± 0.00 ^b^	18.02 ± 0.01 ^j^	2.58 ± 0.02 ^k^	2.71 ± 0.03 ^i^	13.41 ± 0.03 ^b^	32.04 ± 0.13 ^d^	0.26 ± 0.02 ^g^
		AA	4 °C	0.44 ± 0.01 ^e^	20.36 ± 0.02 ^i^	4.72 ± 0.05 ^h^	3.58 ± 0.02 ^h^	2.58 ± 0.02 ^h^	33.74 ± 0.01 ^c^	0.40 ± 0.01 ^b^
			25 °C	0.42 ± 0.00 ^f^	22.41 ± 0.01 ^g^	5.38 ± 0.03 ^f^	4.65 ± 0.01 ^f^	1.64 ± 0.01 ^m^	33.85 ± 0.13 ^c^	0.39 ± 0.03 ^b^
			55 °C	0.38 ± 0.01 ^g^	23.86 ± 0.02 ^f^	5.81 ± 0.01 ^e^	4.92 ± 0.02 ^f^	2.81 ± 0.02 ^f^	32.14 ± 0.13 ^d^	0.37 ± 0.02 ^c^
60	Light	NB	4 °C	0.84 ± 0.00 ^c^	22.73 ± 0.01 ^g^	5.85 ± 0.01 ^e^	4.68 ± 0.01 ^f^	3.16 ± 0.01 ^e^	27.25 ± 0.09 ^i^	0.22 ± 0.01 ^j^
			25 °C	0.80 ± 0.01 ^c^	24.93 ± 0.01 ^d^	6.90 ± 0.05 ^b^	6.59 ± 0.01 ^c^	4.99 ± 0.01 ^d^	27.38 ± 0.13 ^i^	0.20 ± 0.01 ^k^
			55 °C	0.72 ± 0.01 ^d^	15.75 ± 0.01 ^m^	0.32 ± 0.01 ^l^	0.57 ± 0.01 ^k^	15.10 ± 0.01 ^a^	27.24 ± 0.13 ^i^	0.21 ± 0.02 ^j^
		AA	4 °C	0.40 ± 0.01 ^f^	20.30 ± 0.07 ^i^	4.09 ± 0.01 ^j^	3.25 ± 0.02 ^h^	2.81 ± 0.02 ^f^	28.18 ± 0.01 ^h^	0.32 ± 0.00 ^e^
			25 °C	0.36 ± 0.00 ^h^	21.05 ± 0.01 ^h^	4.42 ± 0.05 ^i^	3.88 ± 0.02 ^g^	3.14 ± 0.01 ^e^	28.27 ± 0.13 ^h^	0.31 ± 0.01 ^e^
			55 °C	0.27 ± 0.01 ^j^	24.18 ± 0.06 ^e^	5.69 ± 0.01 ^e^	5.59 ± 0.01 ^d^	2.04 ± 0.03 ^j^	27.15 ± 0.13 ^i^	0.30 ± 0.01 ^e^
	Dark	NB	4 °C	0.86 ± 0.00 ^c^	22.89 ± 0.01 ^g^	6.19 ± 0.01 ^d^	4.94 ± 0.01 ^f^	3.05 ± 0.01 ^e^	28.31 ± 0.09 ^h^	0.25 ± 0.02 ^h^
			25 °C	0.85 ± 0.01 ^c^	25.27 ± 0.01 ^c^	6.92 ± 0.04 ^b^	6.65 ± 0.01 ^b^	4.85 ± 0.01 ^d^	28.41 ± 0.13 ^h^	0.24 ± 0.00 ^i^
			55 °C	0.76 ± 0.01 ^d^	16.45 ± 0.02 ^l^	2.17 ± 0.01 ^k^	1.98 ± 0.02 ^j^	15.01 ± 0.01 ^a^	28.15 ± 0.13 ^h^	0.24 ± 0.02 ^i^
		AA	4 °C	0.42 ± 0.01 ^f^	20.32 ± 0.07 ^i^	4.27 ± 0.01 ^i^	3.41 ± 0.02 ^h^	2.77 ± 0.02 ^g^	29.34 ± 0.01 ^g^	0.36 ± 0.01 ^c^
			25 °C	0.39 ± 0.00 ^g^	21.81 ± 0.01 ^h^	4.94 ± 0.04 ^g^	4.12 ± 0.02 ^g^	3.12 ± 0.01 ^e^	29.51 ± 0.13 ^g^	0.35 ± 0.02 ^d^
			55 °C	0.31 ± 0.01 ^i^	24.20 ± 0.06 ^e^	5.72 ± 0.01 ^e^	5.38 ± 0.02 ^d^	2.21 ± 0.03 ^i^	29.24 ± 0.13 ^g^	0.34 ± 0.02 ^d^

Each value was expressed as mean ± standard deviation (*n* = 3). “a–m” means different letters in the same column have significant differences (*p* < 0.05). “–” means not detected. NB, AA represent non-blanching and continuous aging, hot air-assisted aging cycle, respectively.

## Data Availability

The original contributions presented in the study are included in the article, further inquiries can be directed to the corresponding author.
